# Preoperative Patient-Reported Outcomes in Suspected Low-Grade Glioma: Markers of Disease Severity and Correlations with Molecular Subtypes

**DOI:** 10.3390/jcm10040645

**Published:** 2021-02-08

**Authors:** Dongni Buvarp, Isabelle Rydén, Katharina S. Sunnerhagen, Thomas Olsson Bontell, Tomás Gómez Vecchio, Anja Smits, Asgeir Store Jakola

**Affiliations:** 1 Department of Clinical Neuroscience, Institute of Neuroscience and Physiology, Sahlgrenska Academy, 40530 Gothenburg, Sweden; isabelle.ryden@neuro.gu.se (I.R.); ks.sunnerhagen@neuro.gu.se (K.S.S.); tomasgomezvecchio@gmail.com (T.G.V.); anja.smits@neuro.gu.se (A.S.); asgeir.jakola@vgregion.se (A.S.J.); 2 Department of Neurology, Sahlgrenska University Hospital, 41345 Gothenburg, Sweden; 3 Department of Rehabilitation Medicine, Sahlgrenska University Hospital, 41345 Gothenburg, Sweden; 4 Department of Clinical Pathology and Cytology, Sahlgrenska University Hospital, 41345 Gothenburg, Sweden; thomas.olsson@vgregion.se; 5 Department of Physiology, Institute of Neuroscience and Physiology, University of Gothenburg, Sahlgrenska Academy, 40530 Gothenburg, Sweden; 6 Department of Neuroscience, Neurology, Uppsala University, 75185 Uppsala, Sweden; 7 Department of Neurosurgery, Sahlgrenska University Hospital, 41345 Gothenburg, Sweden

**Keywords:** surveys and questionnaires, quality of life, glioma, fatigue, psychological distress, preoperative, patient-reported outcome measures

## Abstract

This prospective study aims to determine the overall health-related quality of life (HRQoL), functioning, fatigue, and psychological distress preoperatively in patients with suspected diffuse low-grade glioma (dLGG). We were particularly interested if these parameters differed by molecular tumor subtypes: oligodendroglioma, *IDH*mut astrocytoma and *IDH*wt astrocytoma. Fifty-one patients answered self-assessed questionnaires prior to operation (median age 51 years; range 19–75; 19 females [37%]). Thirty-five (69%) patients had *IDH*-mutated tumors, of which 17 were 1p/19q codeleted (i.e., oligodendroglioma) and 18 non-1p/19q codeleted (i.e., *IDH*mut astrocytoma). A lower overall generic HRQoL was associated with a high level of fatigue (r_s_ = −0.49, *p* < 0.001), visual disorder (r_s_ = −0.5, *p* < 0.001), motor dysfunction (r_s_ = −0.51, *p* < 0.001), depression (r_s_ = −0.54, *p* < 0.001), and reduced functioning. Nearly half of the patients reported high fatigue (23 out of 51 patients) and anxiety (26/51 patients). Patients with *IDH*wt had worse generic HRQoL, worse functioning, and more severe fatigue, though differences were not statistically significant between the molecular subtypes. In conclusion, fatigue and anxiety are prominent self-assessed symptoms of patients with suspected dLGG in a preoperative setting, but do not seem to be a reliable method to make assumptions of underlying biology or guide treatment decisions.

## 1. Introduction

Diffuse low-grade gliomas (dLGG) are slow-growing, infiltrative primary brain tumors typically affecting young or middle-aged adults. Patients with suspected dLGG suffer frequent seizures and cognitive deficits, resulting in a negative impact on quality of life [1,2,3,4]. Given its infiltrative and invasive nature, treatment strategies including neurosurgical resection, chemotherapy, or radiation therapy cannot fully eradicate tumor cells, imposing a major challenge to the clinical management in patients with suspected dLGG [5]. The residual tumor is associated with a high risk of postoperative recurrence and malignant progression [6].

The latest WHO classification for dLGG has incorporated molecular markers based on genetic classification [7], such as isocitrate dehydrogenase (*IDH1* or *IDH2*) mutation and codeletion of chromosome arms 1p and 19q, thereby addressing the previous limitations in the histological classification with problematic inter-rater variability and imperfect prediction of outcomes [8,9]. The 2016 WHO classification has been demonstrated to provide a more accurate prognostication and prediction of treatment response [7]. Patients with IDH wildtype (*IDH*wt) LGG show a clinical course similar to primary glioblastoma and significantly shorter survival time than those with *IDH*-mutated (*IDH*mut) tumors of similar malignancy grade [6]. Treatment responses also differ considerably among patients with different molecular profiles [10,11,12].

Patient-reported outcomes (PRO), such as quality of life, fatigue, functioning, and psychological distress, are of clinical importance in dLGG as these measures can be used to determine the patients’ needs. There is a high prevalence of fatigue in patients with dLGG [13,14,15,16,17]. Most studies have been focused on health-related quality of life (HRQoL) and fatigue in a postoperative setting in patients where classification has been based solely on the histological classification [13,16,17]. Little is known about preoperative health conditions and fatigue in patients with suspected dLGG classified by the 2016 WHO classification, and whether the patient-reported symptom burden reflects the underlying biology. As dLGG with and without *IDH*mut have different distributions in the brain [18], and clinical behavior symptoms may also be different between different subtypes [19], PRO may be associated with the molecular subtype of dLGG. In such cases, particular symptoms may act as warning signals and help identify patients at risk for a more rapidly progressing tumor despite radiological appearance of a probable dLGG.

The aim of the study was to explore PRO, including HRQoL, functioning, fatigue, and psychological distress, in patients with suspected dLGG prior to operation, and to explore PRO across different molecular subtypes.

## 2. Materials and Methods

This prospective study was conducted in adult patients (>18 years) for whom initial radiological diagnosis indicated suspected dLGG. Patients were recruited prior to surgical procedures from the Neurosurgical Department at Sahlgrenska University Hospital, Sweden, from January 2017 to December 2019. Patients were identified through the weekly multidisciplinary tumor board (MDTB) meetings, where the possible dLGG diagnosis was noted. In the routine practice both magnetic resonance spectroscopy and more recently also 18F-fluoroethyltyrosine positron emission tomography (FET-PET) were used as part of the diagnostic workup for more challenging cases. In cases with a firm belief that the diagnosis was dLGG, a tissue diagnosis (i.e., biopsy or resection) was recommend upfront (as opposed to “wait and scan”), otherwise, a model for shared decision making was applied [20].

Following MDTB meetings, patients were typically informed by the referring physician about the findings of a suspected dLGG and scheduled for a neurosurgical consultation within two weeks. Within one week after neurosurgical consultation, a formal neuropsychological assessment was made. Prior to this assessment, patients filled out the questionnaires related to HRQoL, fatigue, anxiety, and depression. The neuropsychologist screened the questionnaires for completeness and the answers/symptoms as part of the consultation.

**Figure 1:**
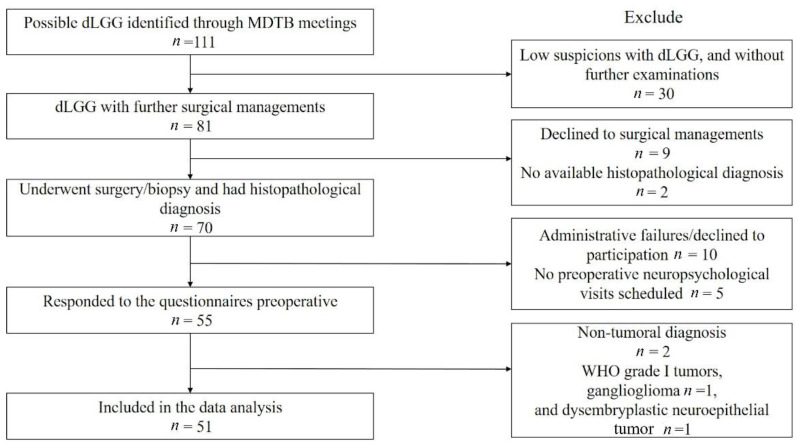
Flow chart of the study sample. MDTB, multidisciplinary tumor board.

Clinical variables were extracted from electronic medical records. Radiological assessments were conducted by an experienced neurosurgeon based on magnetic resonance image using T2-weighted/FLAIR images and the University of California, San Francisco (UCSF)score for grading of eloquence [21]. No restrictions of exclusion criteria were applied on patients with cognitive impairments or communication deficits; however, patients needed to be able to understand and provide informed consent. A flow chart of inclusion for study population is shown in Figure 1. Written informed consent was obtained from all participants prior to the study.

### 2.1. Assessment of Molecular Status

The tumors were classified based on combined histological and molecular findings according to the 2016 WHO classification system [7]. To assess the presence of *IDH* mutations, immunohistochemistry staining for *IDH1* R132H mutant protein was performed. If negative next generation sequencing (NGS) to detect other *IDH1* mutations or *IDH2* mutations were applied. The presence of codeletion of 1p and 19q was evaluated with fluorescence in situ hybridization (FISH).

### 2.2. Assessment of Preoperative Health-Related Quality of Life

Disease-specific HRQoL was assessed using two instruments of European Organization for Research and Treatment of Cancer (EORTC): quality of life questionnaire core-30 (QLQ-C30) and brain cancer-specific quality of life questionnaire (QLQ-BN20). EORTC QLQ-C30 is a 30-item instrument with four response levels that assess functioning, cancer-related symptoms, and global health status [22]. Similar to QLQ-30, QLQ-BN20 is an instrument for assessment of HRQoL and symptom severity, specifically designed for brain cancer patients. QLQ-BN20 consists of four domains for assessing future uncertainty, visual disorder, motor dysfunction, and communication deficit, and seven single items [23]. Each item comprises five response levels (0 corresponds to “not at all”, and 4 corresponds to “very much”).

The generic quality-of-life was assessed using EuroQoL-5-dimension, three levels of response (EQ-5D-3L) together with the associated visual analog scale ranging from 0 to 100 [24]. The results of EQ-5D-3L were transformed to a utility index from −0.594 to 1 [25], where higher scores indicate better health status.

### 2.3. Assessment of Preoperative Fatigue

The 20-item self-assessed multidimensional fatigue inventory (MFI) questionnaire was used to evaluate fatigue in the following five domains in patients with suspected dLGG: general fatigue, physical fatigue, mental fatigue, reduced motivation, and reduced activity [26]. Each domain consists of four items, and each item was assessed in a five-point scale with higher scores indicating more fatigue. MFI has previously been shown to be a valid and reliable instrument for measuring fatigue in patients with cancer [27]. As there was no clear cut-off value previously demonstrated for indicating fatigue level in cancer-related patients, MFI scores were analyzed and presented descriptively as subdomain scores. The fatigue scale of QLQ-C30 was further used to determine a high or low fatigue level in patients with dLGG by using a previously suggested cut-off of 39 scores as the threshold for clinical importance [28]. A score of >39 in the fatigue scale of QLQ-C30 indicates a high level of fatigue.

### 2.4. Assessment of Preoperative Psychological Distress

Psychological distress in patients with suspected dLGG was assessed using the hospital anxiety and depression scale (HADS), a self-assessed questionnaire that consists of two subscales with a total of 14 items. Each scale, one for measuring anxiety and one for depression, contains 7 items. Each item has four response levels (scored 0 to 3) resulting in a total of 21 for each subscale [29]. A score between 8 and 10 suggests mild symptoms or possible disorder, 11–14 moderate symptoms or probable presence of disorder, and 15–21 indicating (moderate to) severe symptom of anxiety or depression [30].

### 2.5. Statistical Analysis

Statistical analysis was performed using IBM SPSS Statistics 26 (IBM Corp., Armonk, NY, USA). Descriptive statistics are presented in percentages, means with standard deviations, or medians with interquartile range, as appropriate.

For the measures of quality of life, QLQ-C30 and QLQ-BN20 were both converted to a continuous index from 0 to 100 according to the scoring manual [31]. A higher score indicates a higher level of corresponding functioning or symptom. Each item in QLQ-C30 and QLQ-BN20 were further dichotomized (“not at all” and “a little” versus “quite a bit and “very much”) for describing distribution of high symptom burden for each molecular subtype. The internal consistency, using Cronbach’s alpha, was 0.75 to 0.91 for all multi-item domains of QLQ-C30, and 0.76 to 0.84 for QLQ-BN20.

Mean scores with standard deviation of MFI are presented to describe different domains of fatigue. Descriptive analysis was also conducted to describe clinical characteristic of patients with a high or low fatigue level that was dichotomized using the fatigue scale of QLQ-C30.

Spearman rank-order correlations were calculated to determine the association between HRQoL, fatigue, psychological distress, QLQ-BN20 domains, QLQ-C30 functioning subscales and items. For group comparisons among different molecular subtypes, Pearson χ^2^ or Fisher’s exact test were used for categorical variables. Independent *t*-test or one-way analysis of variance were used for comparing parametric variables, and the Mann–Whitney U test or the Kruskal–Wallis test for nonparametric variables, as appropriate. A two-tailed *p* < 0.01 was considered statistically significant.

## 3. Results

Fifty-one patients with radiological diagnosis of suspected dLGG answered the questionnaires (median age 51 years; range 19–75; 19 females [37%]) and were included in the data analysis. Patients with suspected dLGG were classified into three categories based on molecular subtypes: patients with *IDH* mutation and 1p/19q codeletion (i.e., oligodendroglioma), patients with *IDH* mutation and non-1p/19q codeletion (i.e., *IDH*mut astrocytoma), and patients with *IDH*wt (i.e., *IDH*wt astrocytoma). Thirty-five of 51 (69%) patients had *IDH*-mutated tumors, and 16 patients (31%) had tumors that were *IDH*wt. Of these 35 patients with *IDH*mut tumors, 17 (47%) had oligodendrogliomas (combined with 1p/19q codeletion) and 18 (53%) had astrocytomas (non-1p/19q codeleted). Details of demographics and clinical characteristic are presented in Table 1.

### 3.1. Preoperative HRQoL and Functioning in Patients with Suspected dLGG and by Molecular Subtype

**Figure 2:**
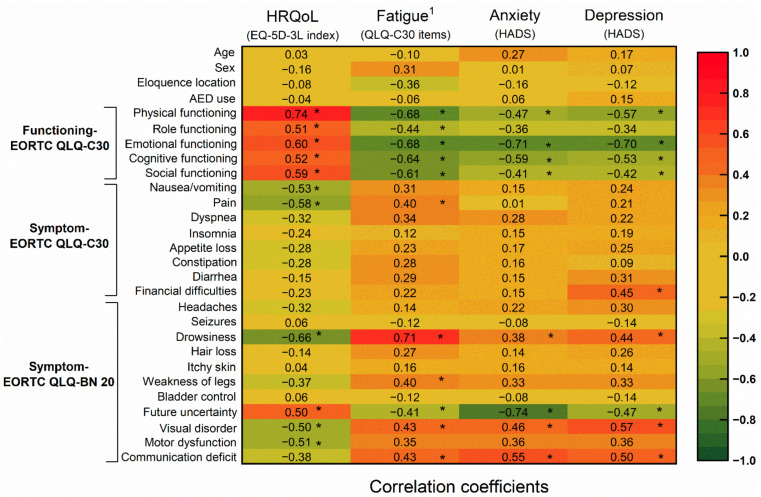
Correlations between patient-reported outcomes, fatigue, and psychological distress in patients with suspected dLGG. * indicate significant values. ^1^ The high or low level of fatigue was classified by using a cut-off value of 39 points in the fatigue scale of QLQ-C30. EORTC, the European Organization for Research and Treatment of Cancer; QLQ-C30, quality of life questionnaire, core-30. QLQ-BN 20, quality of life questionnaire brain cancer module; HADS, hospital anxiety and depression scale; HRQoL, health-related quality of life; EQ-5D-3L, EuroQoL 5-dimension with three responses levels.

Table 2 presents preoperative HRQoL, functioning, and psychological distress in all patients as well as among three molecular subtypes. There was no statistically significant difference in any measures of HRQoL and functioning by *IDH* status of the tumor (Table 2). All aspects of functioning assessed by QLQ-C30, was positively correlated with self-assessed generic HRQoL in patients with suspected dLGG (*p* < 0.001, Figure 2). The generic HRQoL was also significantly correlated with future uncertainty (r_s_ = 0.5, *p* < 0.001), visual disorder (r_s_ = −0.5, *p* < 0.001) and motor dysfunction (r_s_ = −0.51, *p* < 0.001), but not communication deficit (Figure 2). Nausea or vomiting (r_s_ = −0.53, *p* < 0.001), pain (r_s_ = −0.58, *p* < 0.001), and drowsiness (r_s_ = −0.66, *p* < 0.001) were negatively associated with reported generic HRQoL (Figure 2).

**Figure 3:**
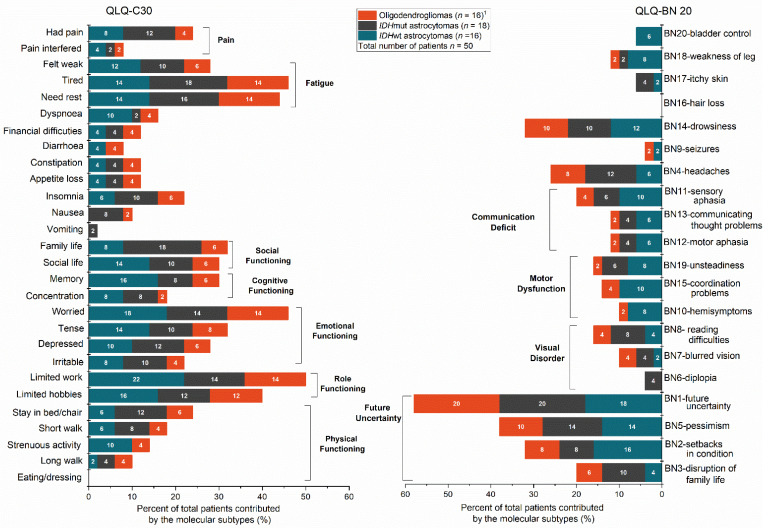
Percentages of patients with “quite a bit” or “very much” symptom burden out of total number of patients in each single item and corresponding domains of QLQ-C30 and QLQ-BN20 are shown. ^1^ Missing data from one patient in the oligodendroglioma group. QLQ, quality of life questionnaire; QLQ-C30, quality of life questionnaire, core-30. QLQ-BN 20, quality of life questionnaire brain cancer module.

The percentage of patients with high symptom burden within each item is presented in Figure 3. The most frequently reported symptoms in patients with suspected dLGG were limited ability to work (50%), feeling worried (46%), feeling tired (46%), and need rest (44%) measured by QLQ-C30. A high frequency of uncertainty regarding the future (58%) was also reported in patients with suspected dLGG assessed by QLQ-BN20. As shown, a higher proportion of patients with *IDH*wt astrocytomas experienced motor dysfunction assessed by QLQ-BN20 compared to other molecular tumor subtypes (Figure 3).

### 3.2. Preoperative Fatigue Based on MFI and the Fatigue Scale of QLQ-C30

**Figure 4:**
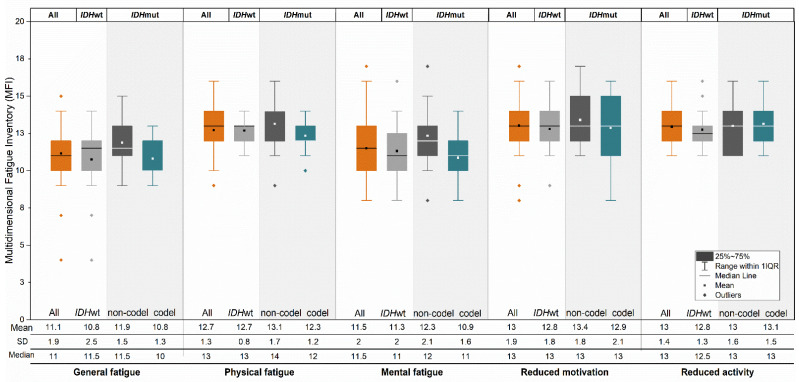
Mean, standard deviation, and median scores for five domains of multidimensional fatigue inventory (MFI) are presented in patients with *IDH*wt (light gray box), or with *IDH*mut combined with 1p/19q codeletion (oligodendrogliomas) in pine green box or *IDH*mut without codeletion (*IDH*mut astrocytomas) in dark grey box, respectively. The 25th and 75th percentiles of scores are indicated by the box, and the range is indicated by the whiskers.

Figure 4 shows the five domains of fatigue, assessed by MFI, and presented for all patients and by molecular subtypes. Patients with suspected dLGG had a lower mean score regarding general and mental fatigue than for other fatigue domains. Patients with *IDH*mut astrocytomas showed the highest scores in general, physical and mental fatigue, and reduced motivation among molecular subtypes (Figure 4); however, there was no statistically significant difference.

Table 3 shows the clinical characteristics and demographics between low and high level of fatigue assessed by QLQ-C30 in patients with suspected dLGG. There were 23 patients (45%) with suspected dLGG that reported high fatigue, and 27 patients (53%) reported a low level of fatigue. A high level of fatigue was significantly correlated with a lower overall generic HRQoL (r_s_ = −0.49, *p* < 0.001). In addition, a high level of fatigue was associated with substantial visual disorder (r_s_ = 0.43, *p* = 0.002), communication deficit (r_s_ = 0.43, *p* = 0.002), weakness of legs (r_s_ = 0.40, *p* = 0.004), drowsiness (r_s_ = 0.71, *p* < 0.001), pain (r_s_ = 0.40, *p* = 0.004), and reduced functioning (Figure 2).

### 3.3. Preoperative Anxiety and Depression

In total, 26 patients with suspected dLGG (51%) had anxiety, and 10 patients (20%) had depression. Moderate anxiety was reported by 7 patients (14%), and there were 2 patients (4%) with both severe anxiety and depression. Anxiety and depression were both significantly associated with a high level of fatigue assessed by QLQ-C30 (r_s_ = 0.44, *p* = 0.002). Patients with depression had a lower overall generic HRQoL (r_s_ = −0.54, *p* < 0.001).

**Figure 5:**
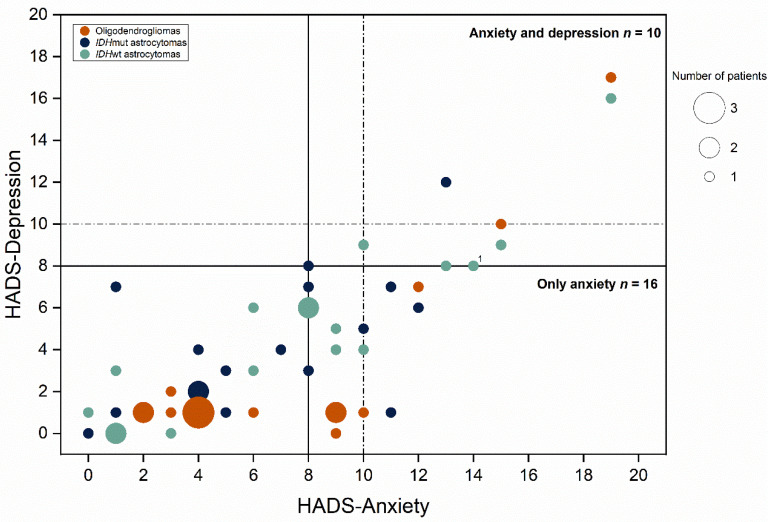
Scatter plot of self-reported anxiety and depression assessed using hospital anxiety and depression scale (HADS). A score between 8 (indicates in a solid line) and 10 (indicates in a dash line) in HADS suggests mild symptoms or possible disorder. A score > 10 in HADS indicate moderate or moderate to severe symptoms of anxiety or depression. Ten patients considered to have both anxiety and depression and 16 patients were considered to be have anxiety. ^1^ Two patients had the same score in HADS, one in the oligodendroglioma group and another one in the *IDH*wt astrocytoma group.

Figure 5 shows the number of patients by molecular subtypes who had anxiety or depression measured using HADS. Percentages of reported anxiety were similar among patients with *IDH*wt and with *IDH*mut astrocytomas (9 patients [18% of total patients]). No statistically significant difference was found in anxiety or depression between the molecular subtypes. There were 31% of patients with *IDH*wt reported depression, while those with *IDH*mut astrocytomas reported in 18% and patients with oligodendrogliomas in 11%.

## 4. Discussion

In this prospective study, we found that fatigue and anxiety are prominent symptoms in patients with suspected dLGG prior to operation. Importantly, high levels of preoperative fatigue and depression were significantly associated with a lower self-assessed generic HRQoL. Patients with *IDH*wt tumors, in general, tended to have poorer generic HRQoL, worse functioning, and more severe fatigue. However, no significant difference in fatigue, anxiety, depression, and generic or disease-specific HRQoL was found between molecular subtypes in a preoperative setting.

The study group as a whole showed lower generic HRQoL and higher fatigue than the Swedish general population, as measured by EQ-5D-3L (reference interval 0.79 to 0.89) [32] and MFI scores (reference interval 7.7 to 10), respectively [33]. This is in line with previous studies regarding preoperative fatigue and HRQoL in patients with suspected dLGG [2,16]. Additionally, a negative association between lower HRQoL and higher fatigue was also in accordance with earlier findings [14,15].

The prevalence of high-level fatigue (45%) in this study is comparable to prior studies where the similar cut-off above 39 was used [16]. Unexpectedly, as seizures are common in patients with LGG [34], only few patients reported seizure-related symptoms, and seizures were found to not be statistically significantly associated with a high level of fatigue. This may be explained by the fact that approximately 70% of patients in the present study had antiepileptic drugs preoperatively and evaluation was performed early in the disease course, when good seizure control was obtained. This time aspect naturally influences the patient-reported seizure related-symptom burden, and is particularly important to consider since the EORTC questionnaire assesses symptoms during the past week.

No statistically significant difference was found between molecular subtypes in any self-assessed well-being and other symptoms. Thus, symptom monitoring or well-being of patients or distress is not reliably associated with the underlying biology of the tumor at the time of diagnosis. This is perhaps not surprising since symptom monitoring is not a reliable predictor of malignant transformation, but it is still a reminder that the current well-being of patients is not an indicator of an indolent lesion [35]. However, symptom monitoring is still necessary in order to assist in identifying the needs of patients and to prevent early worsening of any disabilities. In the preoperative setting, we need better radiological measures or access to minimal invasive techniques (i.e., liquid biopsies) to establish diagnosis with more certainty, as symptom monitoring alone is insufficient [36].

In this study, patients with suspected dLGG showed anxiety without clearly reported depression prior to operation. Higher median scores of anxiety and depression in patients with suspected dLGG was found compared to the Swedish population (reference interval 3 to 4) [37]. It is common that patients with brain tumor diagnosis experience increased level of anxiety in addition to tumor-related fatigue. Anxiety among patients with suspected dLGG still seems to be undertreated in the preoperative setting despite earlier studies emphasizing this matter [38]. A psychological assessment prior to operation is often beneficial for recognizing any potential long-term anxiety or depression, and to apply an appropriate antidepressant drug therapy in moderation.

The strengths of the present study are that PRO were evaluated preoperatively and classified based on molecular markers rather than the histological classification. This may provide a more accurate picture of patients with suspected dLGG for predicating prognosis and outcomes as well as increasing clinical interpretations for treatment-related effects. The study findings provided comprehensive preoperative data on overall health condition, ranging from HRQoL, functioning, and fatigue to psychological distress in patients with suspected dLGG, which can contribute to future studies aiming to address those areas with high symptom burden. Limitations of the study are the relatively small sample size, which allows for a solely descriptive interpretation without adjustment of confounding factors (e.g., age and gender). Although consistent trends were seen across molecular subgroups, they were not statistically significant or of a magnitude that limits the clinical usefulness of screening of symptoms as such for highlighting underlying tumor behavior. Moreover, patient self-reported outcomes were used that have the advantages of providing subjective insights only known to the patients themselves and without influence by the clinicians. Self-reported outcomes are, however, subjective and may be prone to recall- and emotional bias.

## 5. Conclusions

Fatigue and anxiety are prominent symptoms in patients with suspected dLGG already in the preoperative setting. A high level of fatigue and reduced functioning was associated with a lower HRQoL. There was no statistically significant difference for any PRO measures between molecular subtypes, indicating that clinical presentation of symptoms and well-being patients in a pre-operative setting is not a reliable method to make assumptions of underlying biology or guide treatment decisions.

## Figures and Tables

**Table 1:** The demographics and clinical characteristic of the patients with suspected dLGG based on the molecular subtypes.

Characteristic	All (*n* = 51)	*IDH*wt Astrocytomas (*n* = 16)	*IDH*mut Astrocytomas (*n* = 18)	Oligodendrogliomas (*n* = 17)
Age, mean (SD)	49 (14)	56 (12)	41 (14)	50 (13)
Sex, No. (%)				
Male	32 (63)	11 (69)	11 (61)	10 (59)
Female	19 (37)	5 (31)	7 (39)	7 (41)
Tumor location, No. (%)				
Frontal	23 (45)	7 (47)	6 (33)	10 (59)
Temporal	12 (24)	5 (33)	6 (33)	1 (6)
Parietal	5 (10)	0 (0)	4 (22)	1 (6)
Insula	6 (12)	1 (7)	2 (11)	3 (18)
Others ^1^	5 (10)	3 (19)	0 (0)	2 (12)
Lateralization, No. (%)				
Left	24 (47)	8 (53)	12 (67)	4 (24)
Right	25 (49)	6 (40)	6 (33)	13 (76)
Bilateral/midline	2 (4)	2 (13)	0 (0)	0 (0)
Multifocal lesions, No. (%)	8 (16)	5 (31)	1 (6)	2 (12)
Eloquent location, No. (%)	35 (69)	12 (75)	13 (72)	10 (59)
Histologic type and grade, No. (%)				
Grade II	31 (61)	8 (50)	14 (78)	9 (53)
Grade III	16 (31)	6 (38)	2 (11)	8 (47)
Grade IV	4 (8)	2 (12)	2 (11)	0 (0)
AED treatment, No. (%)	35 (69)	13 (81)	12 (67)	10 (59)
Antidepressants, No. (%)				
SSRI	3 (6)	1 (7)	1 (9)	1 (6)
Tricyclic antidepressants	2 (4)	0 (0)	1 (9)	1 (6)
Anxiolytic/Hypnotics, No. (%)				
Benzodiazepines	7 (14)	3 (20)	2 (11)	2 (12)
Zopiclone	22 (43)	8 (50)	7 (39)	7 (41)
Others ^2^	3 (6)	0 (0)	2 (11)	1 (6)

^1^ gyrus cingula *n* = 1; gliomatosis *n* = 2; thalamus *n* = 2; ^2^ propiomazine *n* = 1; hydroxyzine *n* = 2; IDH, isocitrate dehydrogenase; AED, antiepileptic drug; SSRI, selective serotonin reuptake inhibitors.

**Table 2:** Preoperative patient-reported outcomes in patients with suspected dLGG based on molecular markers.

	All (*n* = 51)	*IDH*wt Astrocytomas (*n* = 16)	*IDH*mut Astrocytomas (*n* = 18)	Oligodendrogliomas ^1^ (*n* = 17)	*p*-Value ^2^	*p*-Value ^3^
Functioning—EORTC QLQ-C30						
Physical functioning	81 (20)	76 (17)	82 (18)	84 (25)	0.16	0.09
Role functioning	55 (35)	42 (38)	59 (29)	64 (38)	0.18	0.07
Emotional functioning	63 (27)	58 (24)	61 (28)	71 (27)	0.30	0.28
Cognitive functioning	68 (29)	57 (30)	69 (25)	77 (29)	0.10	0.06
Social functioning Symptom domains—EORTC QLQ-BN20	66 (31)	62 (28)	63 (34)	75 (32)	0.26	0.26
Future uncertainty	59 (25)	52 (26)	61 (25)	64 (23)	0.39	0.19
Visual disorder	13 (20)	10 (17)	19 (26)	8 (14)	0.39	0.57
Motor dysfunction	19 (25)	30 (30)	14 (18)	13 (24)	0.14	0.06
Communication deficit	20 (26)	28 (31)	18 (23)	13 (23)	0.10	0.06
Psychological distress ^4^						
HADS—anxiety, median (IQR)	8 (4–10.5)	9 (4–12)	7 (4–10)	9 (4–11)	0.62	0.68
HADS—depression, median (IQR)	3 (1–7)	5 (2–8)	4 (1–7)	1 (1–7)	0.44	0.29
HRQoL						
EQ-5D-3L index—overall health ^5^	0.67 (0.3)	0.61 (0.25)	0.67 (0.3)	0.73 (0.33)	0.34	0.14
EQ-VAS ^5^	61 (27)	59 (33)	65 (23)	58 (26)	0.77	0.98
QLQ-C30—global health status	55 (24)	50 (25)	55 (24)	61 (25)	0.41	0.41

Data are mean ± standard deviation. ^1^ Missing data in QLQ-C30 and QLQ-BN20 for one patient in the oligodendroglioma group. ^2^ Group comparisons were conducted between 3 molecular subtypes. Pearson χ^2^, 1-way analysis of variance, or the Kruskal–Wallis test was used as appropriate. ^3^ Group comparisons were conducted between *IDH* mutation and *IDH* wildtype. Pearson χ^2^, Fisher’s exact test, independent *t*-test, or the Mann–Whitney U test was used as appropriate. ^4^ Data in HADS were available in 49 patients. ^5^ Data were reported in 47 patients in EQ-5D-3L, and EQ-VAS was available in 46 patients. EORTC, the European Organization for Research and Treatment of Cancer; QLQ-C30, quality of life questionnaire, core-30. QLQ-BN 20, quality of life questionnaire brain cancer module; HADS, hospital anxiety and depression scale; IQR, interquartile range; HRQoL, health-related quality of life; EQ-5D-3L, EuroQoL 5-dimension with three responses levels; EQ-VAS, EuroQoL visual analog scale.

**Table 3:** The demographics and clinical characteristic of the patient with suspected dLGG in low and high level of fatigue. ^1^

Characteristic	High Fatigue (*n* = 23)	Low Fatigue (*n* = 27)	*p*-Value ^2^
Age, mean (SD)	47 (15)	50 (14)	0.51
Sex, No. (%)			0.03
Male	11 (42)	21 (78)	
Female	12 (52)	6 (22)	
Tumor location, No. (%)			0.81
Frontal	11 (48)	12 (44)	
Temporal	6 (26)	6 (22)	
Parietal	1 (4)	3 (11)	
Insula	2 (9)	4 (15)	
Others ^3^	3 (13)	2 (7)	
Lateralization, No. (%)			0.14
Left	9 (39)	15 (56)	
Right	14 (61)	10 (37)	
Bilateral	0 (0/0)	2 (7)	
Multifocal lesions, No. (%)	2 (9)	6 (22)	0.26
Eloquent location, No. (%)	12 (52)	23 (85)	0.02
AED treatment, No. (%)	15 (65)	19 (70)	0.70
Molecular subtypes, No. (%)			0.56
*IDH*wt astrocytomas	9 (39)	7 (26)	
*IDH*mut astrocytomas	6 (26)	10 (37)	
Oligodendroglioma	8 (35)	10 (37)	

Data were available in 50 patients. ^1^ The high or low level of fatigue was classified by using a cut-off value of 39 points in the fatigue scale of QLQ-C30. ^2^ Pearson χ^2^, Fisher’s exact test, independent *t*-test, and Mann–Whitney U test were used as appropriate. ^3^ gyrus cingula *n* = 1; gliomatosis *n* = 2; thalamus *n* = 2; SD, standard deviation; IDH, isocitrate dehydrogenase; AED, antiepileptic drug.

## Data Availability

According to the Swedish regulations shown in https://etikprovning.se/for-forskare/ansvar/ (accessed on 7 February 2021), the complete dataset cannot be made publicly available for ethical and legal reasons. Researchers can request access to the data upon reasonable request by emailing the principal investigator at asgeir.jakola@vgregion.se.
